# Inflammatory Myofibroblastic Bladder Tumor in a Patient with Wolf-Hirschhorn Syndrome

**DOI:** 10.1155/2013/675059

**Published:** 2013-08-18

**Authors:** Antonio Marte, Paolo Indolfi, Carmine Ficociello, Daniela Russo, Matilde Oreste, Gaetano Bottigliero, Giovanna Gualdiero, Ciro Barone, Elena Vigliar, Cristiana Indolfi, Fiorina Casale

**Affiliations:** ^1^Pediatric Surgery, Second University of Naples, Largo Madonna delle Grazie, 80138 Naples, Italy; ^2^Pediatric Oncology Service, Pediatric Department, Second University of Naples, Italy; ^3^Pathology Department, Federico II University of Naples, Italy

## Abstract

Inflammatory myofibroblastic tumor (IMT) is a rare neoplasm described in several tissues and organs including genitourinary system, lung, head, and neck. The etiology of IMT is contentious, and whether it is a postinflammatory process or a true neoplasm remains controversial. To our knowledge, we report the first reported case of IMT of urinary bladder in a pediatric patient with Wolf-Hirschhorn (WHS). We also review the literature about patients with associated neoplasia.

## 1. Introduction

IMT is a rare neoplasm usually seen in children and adolescents, mostly occurring between 2–16 years of age. Females are affected slightly more commonly than males. It is also known as cellular inflammatory pseudotumor, plasma cell granuloma, and inflammatory fibrosarcoma and is composed of spindle cells with associated inflammatory cells infiltrate [1]. This type of tumor has been described in several organs and anatomical sites including genitourinary system where the tumor usually originates in the bladder, but it has also been reported in the kidney, urethra, prostate, ureter, and *rete testis* [2]. The etiology of IMT, its behavior, and its cell of origin remain matters of debate [1]. Originally considered a lesion with a benign clinical course, it is now clear that IMT can have an aggressive behavior and, occasionally, an unfavorable prognosis [3]. For this reason, it is important to differentiate this lesion from sarcoma for therapeutic management, and this can be difficult both clinically and histologically [3]. To gain more knowledge about this rare tumor, we reported a case of IMT of the urinary bladder in a girl with WHS.

## 2. Case Report

**Figure 1:**
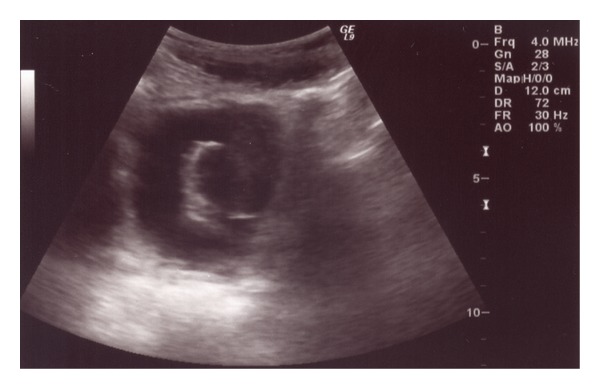
Preoperative US shows a mass arising from the left bladder wall of about 50 × 40 mm.

**Figure 2:**
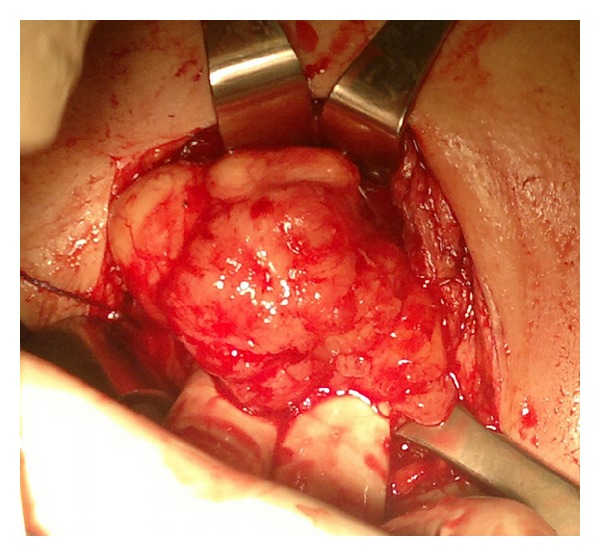
Intraoperative view of the bladder mass.

**Figure 3:**
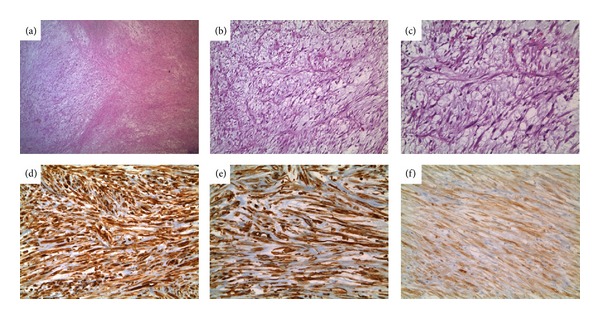
(a) A panoramic view of the inflammatory myofibroblastic tumor: note the cellular proliferation of spindled cells (H&E ×100). (b)-(c) Vesicular nuclei and eosinophilic cytoplasms, organized in a fascicular growth pattern ((b) H&E, ×150; (c) H&E ×200). (d) A widespread immunoreactivity for vimentin (immunoperoxidase stain for vimentin, ×200). (e) A positivity for smooth muscle actin (SMA) (immunoperoxidase stain for SMA, ×200). (f) A diffuse staining for anaplastic lymphoma kinase (ALK) (immunoperoxidase stain for ALK, ×200).

A previously healthy 8-year-old female, with WHS, was admitted to our clinic in February 2012 for a persistent abdominal pain and macroscopic hematuria. Abdominal ultrasound revealed a multilobated tumor in the bladder adhering to the left bladder wall. A computerized tomography (CT) scan of abdomen confirmed these findings, and a solid mass (approximate size 5 × 4.5 cm) infiltrating the dome and the left bladder wall not extending to perivesical tissues nor lymph node enlargement was revealed. Due to ultrasonographic (Figure 1) and CT scan features (large base, poor vascularization, multilobated appearance, and size >4 cm), the patient underwent cystoscopic multiple biopsies. Histopathology revealed a spindle cell lesion with mixed inflammatory cells in the background. According to histopathology and immunohistochemical characteristics, a provisional diagnosis of IMT was made. Because of the size of the tumor, a complete transurethral resection was not technically possible and a chemotherapy or radiotherapy was not justified in the absence of histological malignant tissue, so an open tumor excision was performed. A Pfannenstiel incision was made, and the anterior bladder wall was opened. A large white polypoid mass occupying almost the entire cavity, infiltrating the dome and the left bladder wall, was found. The mass, with a cuff of normal bladder, was excised protecting the ipsilateral ureter with a 4CH ureteral sund. A foley catheter was left in place for three day, and the girl was discharged on day four. The postoperative course was regular, except for the urgency that was treated with oxybutynin. On gross examination, the mass measured 6.5 × 5 × 4.5 cm and was lobulated and firm, with small hemorrhagic areas and a gray-white cut surface containing focal pale yellow areas (Figure 2). There was no evidence of necrosis. The histological evaluation of the mass showed a lesion consisting of spindle cells with vesicular nuclei and eosinophilic cytoplasms, organized in a fascicular growth pattern. Sometimes cells showed a plump appearance with polygonal nuclei and evident nucleoli. Inflammatory cells, mainly plasma cells, were also present. The background showed a myxoid appearance with many blood vessels. Mitotic figures were rare; there was no evidence of necrosis and infiltration along the muscular wall. The resection margins were clear. Immunohistochemically, the tumour cells showed cytoplasmatic positivity for vimentin, smooth muscle actin (SMA), anaplastic lymphoma kinase (ALK), and focal positivity for desmin. Positivity for CK AE1/AE3 was observed in about 15% of the cells. Tumour cells were negative for myoglobin, CD34, and S100 (Figure 3). After an uneventful postoperative recovery, thirteen months after surgery, the patient is alive and well, without late complications.

## 3. Discussion

IMT is an uncommonly benign tumor of unknown neoplastic potential, characterized by proliferation of myofibroblastic spindle cells and inflammatory elements [1]. Originally, IMT was described in the lung, but it has been reported in a variety of extrapulmonary sites, and it is most frequent in the bowel, mesentery, and genitourinary tract [2]. Among the genitourinary organs, the bladder is more commonly affected. Patients with IMT of the urinary tract usually present with painless macroscopic hematuria but may also have lower urinary tract symptoms. Sometimes a palpable mass that mimics a malignancy may be the clinical presentation of this lesion [3]. In our case, the patient complained of abdominal pains and macroscopic hematuria. The size of the reported IMT ranged from 0.4 to 40 cm, although the typical bladder lesion is less than 2 cm and is described as having a circumscribed, solitary or multinodular, gray-white or yellow, fascicular or myxoid appearance [4]. The etiology of IMT is contentious, and whether it is a postinflammatory process or a true neoplasm remains controversial. Proponents for postinflammatory or immunologic processes advocate the association of IMT and infectious agents such as mycobacteria, corynebacteria, Epstein Barr virus, and human herpes virus, as well as prior histories of surgery, trauma, and steroid usage [4, 5]. In contrast, proponents for neoplasm advocate the aggressive behavior, cytogenetic clonality, and the rare possibility of distant metastasis [6]. In our patient, there was no evidence of related infection or traumatic episode. IMT presents morphologic, immunophenotypic, endoscopic, and radiologic overlap with malignant spindle cells tumors of the urinary bladder, and differentiation from these tumors may be difficult [7]. Both epithelial and myogenic markers can be expressed in IMT and this coexpression may lead to a misdiagnosis of sarcomatoid carcinoma, leiomyosarcoma, and rhabdomyosarcoma [8–10]. Recently, a clonal aberration in the short arm of chromosome 2 (region p21–p23) has been reported in approximately 50% of IMT [11]. Chromosome 2p23 is the genetic location of a tyrosine kinase receptor known as anaplastic lymphoma kinase (ALK), whose expression is normally restricted to the central nervous system or results from chromosome translocation. Several ALK fusion partners have been identified [12]. An overexpression of anaplastic-lymphoma kinase (ALK-1) in many of IMT confirms the neoplastic nature of these lesions [12]. The chromosomal rearrangements that involve the ALK gene locus are located in the chromosome 2p23 with other partner genes in chromosome 1 at 17q23 or in chromosome 19 at 19p13. The positivity for ALK-1 in IMT of urinary bladder by immunohistochemistry ranges from 33 to 89%, whereas in leiomyosarcoma and sarcomatoid urothelial carcinoma has not been reported. This suggests that ALK-1 immunohistochemical studies may be useful in the differentiation of IMT from other spindle cell lesion in the urinary bladder [11]. The differentiation between IMT and malignant lesion is very important for both prognosis and therapeutic management. In fact, IMT can be managed with a bladder-preserving approach, that is, transurethral resection (TUR) or partial cystectomy [12]. The Wolf-Hirschhorn syndrome (WHS), also known as chromosome deletion Dillan 4p syndrome, is a rare genetic syndrome with a characteristic phenotype resulting from a partial deletion of chromosomal material of the short arm of chromosome 4. The incidence of this condition is rare and is estimated to be approximately one in 50,000 births [13]. The most common characteristic clinical signs include a distinct craniofacial phenotype (microcephaly, micrognathia, short philtrum, ocular hypertelorism, and dysplastic area), growth and mental retardation, muscle hypotonia, urinary tract malformations (renal agenesis, cystic dysplasia/hypoplasia, oligomeganephronia, bladder exstrophy, and obstructive uropathy), and congenital heart defects [14]. About 87% of cases represent a de novo deletion, usually on the paternal chromosome, while about 13% are inherited from a parent with a chromosome translocation. Severity of symptoms and expressed phenotype are based on the amount of genetic material deleted that always include two minimal critical regions for WHS (WHSCR-1 and -2) [15, 16]. Our patient presented typical phenotypic characteristics with associated bilateral congenital cataract and right renal pyelectasis. To our knowledge, this is the first reported case of IMT of bladder urinary in a pediatric patient with WHS. A case of multiple hemangiomas, one case of cutaneous T-cell Lymphoma in a 21 year-old-male, and two cases of myelodysplastic syndrome in children are only reported [17, 18]. In our case, karyotype analysis of peripheral blood was normal as test fish of chromosome 4. Further studies made it possible to find cryptic rearrangements involving WHSCR. This result is very important considering that patients with WHS without cryptic deletion of WHSCR are rare. Approximately, 25% of patients with the WHS phenotype have either an unbalanced translocation involving chromosome 4p or an unusual genetic makeup such as a mosaicism of 4p [14]. Several candidate genes for the WHS phenotype have been proposed: the Wolf-Hirschhorn candidate 1 (WHSC-1) and WHSC-2 genes that encode for at least five proteins that function as histone methyltransferases; a fourth gene is fibroblast growth factor receptor 3 (FGFR 3) that is a member of the tyrosine kinase receptor family involved in mitogenesis and angiogenesis [19]. It is possible that one or more of the protein encoded by this genes may play a role in the development of neoplastic lesions. An adult patient with BCR/ABL negative myeloproliferative syndrome had a t(4;14)(p16;q24) translocation; in several studies, translocations involving the WHSC-1 gene have been associated with multiple myeloma [15, 16]. Cytogenetic studies in these patients lend to support the hypothesis that a number of genes potentially relevant to tumorogenesis map to chromosome 4p16. According to the results of these studies, it can be assumed that in our case the cryptic rearrangements involving WHSCR may be involved in IMT development. 

Although we are unable to identify a relationship between WHS and tumor, there is evidence that mutations of the distal portion of chromosome 4p may contribute to tumorogenesis. Continued monitoring of patients with WHS and further characterization of the protein encoded in the WHS critic region of chromosome 4 may help to determine whether patients with WHS have an increasing risk of developing a tumor.
